# A Large Congenital Pulmonary Airway Malformation Mimicking a Cystic Thoracic Mass in an Infant: A Case Report

**DOI:** 10.7759/cureus.112915

**Published:** 2026-07-18

**Authors:** Amani A Alharbi, Najla M Al Sudairy

**Affiliations:** 1 College of Medicine, Ibn Sina National College for Medical Studies, Jeddah, SAU; 2 Family Medicine, National Guard Health Affairs, Jeddah, SAU

**Keywords:** congenital cystic lung lesion, congenital lung anomaly, congenital pulmonary airway malformation, cystic lung disease, infant pulmonary malformation, lobectomy, mediastinal shift, multicystic lung mass, pediatric thoracic surgery, recurrent respiratory infection

## Abstract

Congenital pulmonary airway malformation (CPAM) is a rare developmental anomaly of the lung that can present with a wide spectrum of clinical manifestations, ranging from asymptomatic incidental findings to severe respiratory compromise during infancy. We present the case of a one-year-old male infant with a giant multicystic pulmonary lesion involving the right upper and middle lobes, causing significant mediastinal shift and respiratory symptoms. The patient presented with recurrent episodes of lower respiratory tract infection, persistent cough, and progressive shortness of breath. Clinical examination revealed reduced air entry over the right upper and middle lung fields. Chest imaging demonstrated a large multicystic lesion almost completely replacing the right upper and middle lobes. CT of the chest revealed a purely multicystic mass lesion without a visible solid component, abnormal vasculature, or communication with the bronchial system. The lesion produced a significant mass effect with a contralateral mediastinal shift. Based on the clinical and radiological findings, CPAM was considered the most likely diagnosis. The patient underwent surgical resection with right upper and middle lobectomy. Histopathological examination confirmed the diagnosis of CPAM. The postoperative course was uncomplicated with resolution of respiratory symptoms and satisfactory clinical recovery during follow-up. This case highlights the importance of considering CPAM in infants presenting with recurrent respiratory symptoms and complex cystic lung lesions. Large lesions may remain undiagnosed until infancy and can produce significant anatomical distortion through compression of adjacent lung tissue and mediastinal displacement. Detailed radiological assessment is essential for characterization of the lesion, exclusion of vascular anomalies, and operative planning. Early recognition and appropriate surgical management of symptomatic CPAM can provide excellent outcomes and prevent potential complications associated with persistent abnormal lung tissue.

## Introduction

Congenital pulmonary airway malformation (CPAM), previously known as congenital cystic adenomatoid malformation, is a rare developmental anomaly of the lower respiratory tract characterized by abnormal branching morphogenesis of the terminal bronchioles, resulting in cystic and adenomatous replacement of normal pulmonary parenchyma. CPAM represents one of the most common congenital lung malformations, with an estimated incidence ranging from 1 in 10,000 to 1 in 35,000 live births [1,2]. Clinical presentation is highly variable and depends on the size of the lesion, the degree of pulmonary compression, and the presence of associated complications [2,3]. While many lesions are detected during routine prenatal ultrasonography, others remain asymptomatic until infancy or childhood, presenting with recurrent respiratory tract infections, persistent cough, respiratory distress, or, less commonly, spontaneous pneumothorax [1-4]. Cross-sectional imaging, particularly contrast-enhanced CT, plays a pivotal role in delineating lesion morphology, assessing vascular anatomy, and distinguishing CPAM from other congenital cystic lung lesions [1,4].

The differential diagnosis of multicystic pulmonary lesions in infants includes pulmonary sequestration, hybrid lesions, congenital lobar overinflation, bronchogenic cysts, and cystic pleuropulmonary blastoma, making accurate radiological and histopathological evaluation essential for appropriate management [4-7]. Surgical resection remains the standard treatment for symptomatic patients and is also advocated by many centers for asymptomatic lesions because of the risks of recurrent infection, progressive enlargement, pneumothorax, and the potential, albeit rare, for malignant transformation [1,5]. We report the case of a one-year-old infant with a giant multicystic lesion replacing the right upper and middle lobes, producing significant contralateral mediastinal shift and respiratory symptoms. The case highlights the diagnostic challenges, radiological characteristics, surgical management, and favorable postoperative outcome associated with this uncommon congenital pulmonary malformation.

## Case presentation

A 12-month-old previously healthy male infant was referred to our tertiary pediatric surgery service for evaluation of recurrent respiratory symptoms and an abnormal chest radiograph. According to his parents, he had experienced three episodes of lower respiratory tract infection during the preceding four months, each characterized by nonproductive cough, tachypnea, low-grade fever, and reduced oral intake. The episodes had been managed conservatively with oral antibiotics prescribed at local healthcare facilities, with only temporary improvement. During the two weeks before presentation, the child developed progressive shortness of breath during feeding and play, accompanied by an intermittent dry cough but without cyanosis, hemoptysis, or significant fever. There was no history of choking episodes, foreign body aspiration, wheezing suggestive of asthma, contact with individuals with tuberculosis, or recent travel. Antenatal history was unremarkable, with routine prenatal ultrasonography reported as normal. The patient was born at term via spontaneous vaginal delivery with an uncomplicated perinatal course. His developmental milestones were age-appropriate, immunizations were up to date, and there was no history of previous surgery or congenital anomalies. Family history was negative for congenital pulmonary disease, cystic fibrosis, or childhood malignancy.

On arrival, the patient appeared alert but mildly tachypneic. His temperature was 36.8°C, heart rate was 132 beats/min, respiratory rate was 42 breaths/min, blood pressure was 92/56 mmHg, and oxygen saturation was 95% while breathing room air. Growth parameters were within normal percentiles for age. Mild subcostal and intercostal retractions were noted without nasal flaring. Chest inspection demonstrated reduced expansion of the right hemithorax. Palpation revealed slight right-sided reduction in chest excursion, while tracheal deviation was difficult to appreciate clinically. Percussion demonstrated relative hyperresonance over the right upper chest. Auscultation revealed markedly diminished breath sounds over the right upper and middle lung zones with preserved air entry at the right lung base. The left lung demonstrated normal vesicular breath sounds without adventitious sounds. Cardiovascular examination showed normal heart sounds with the point of maximal impulse displaced slightly toward the left hemithorax. The abdomen was soft without hepatosplenomegaly, and the remainder of the systemic examination was unremarkable.

Initial laboratory investigations demonstrated a hemoglobin concentration of 11.6 g/dL, white blood cell count of 11.9 × 10⁹/L with mild neutrophilic predominance, platelet count of 368 × 10⁹/L, and C-reactive protein of 9 mg/L. Serum electrolytes, renal function tests, liver function tests, coagulation profile, and serum lactate were within normal limits. Venous blood gas analysis revealed a pH of 7.39, pCO₂ of 40 mmHg, bicarbonate of 24 mmol/L, and no evidence of respiratory acidosis. Blood cultures obtained on admission showed no bacterial growth.

**Figure 1:**
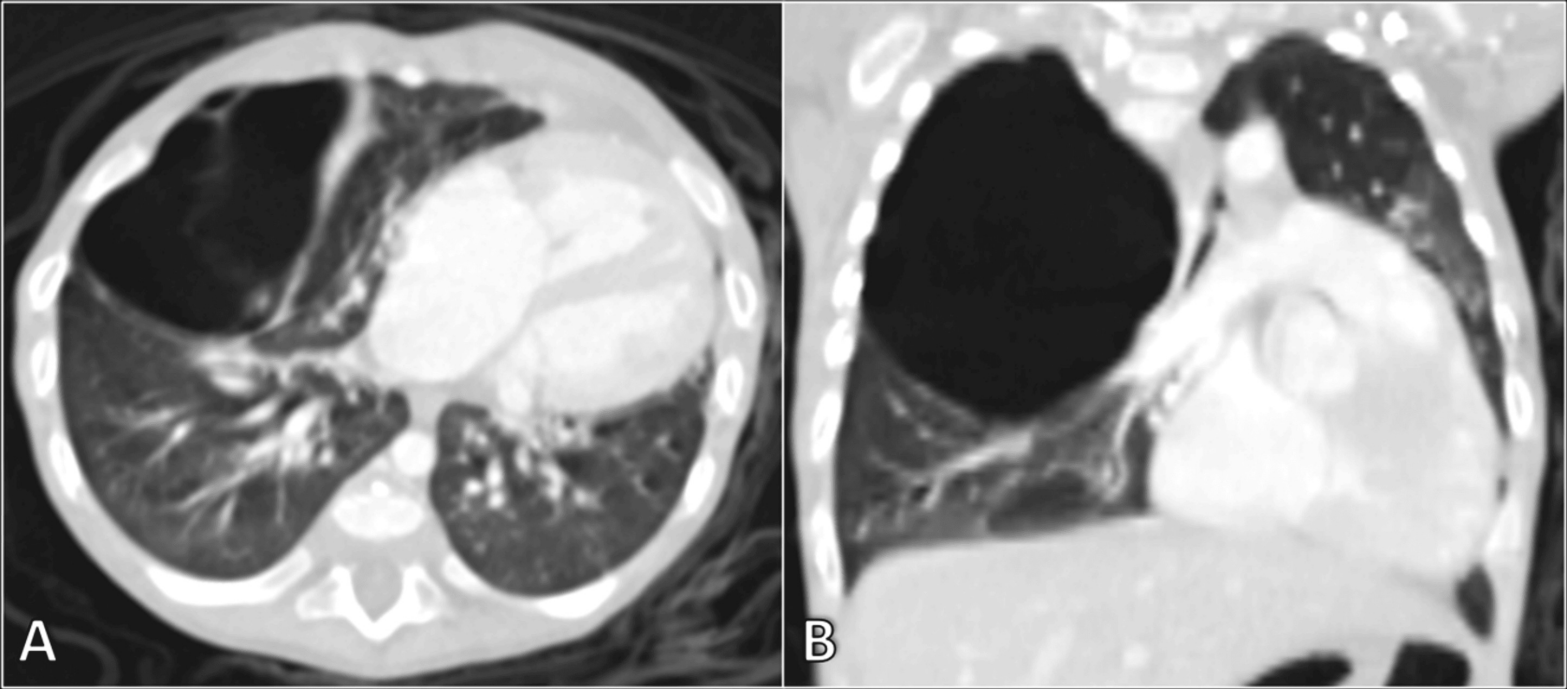
Contrast-enhanced CT images of the chest demonstrating extensive congenital pulmonary airway malformation. (A) Axial CT image showing a large multicystic lesion occupying almost the entire right upper and middle lobes, with multiple variable-sized cystic spaces replacing the normal pulmonary parenchyma. No solid soft tissue component or abnormal vascular structures are identified. The lesion produces significant mass effect with compression of adjacent lung tissue and contralateral mediastinal shift. (B) Coronal CT reconstruction demonstrating the cranio-caudal extent of the multicystic lesion involving the right upper and middle lobes, with marked displacement of the mediastinal structures toward the left side. No communication with the bronchial system or associated anomalous systemic arterial supply is visualized.

Chest radiography demonstrated a large multiloculated hyperlucent lesion occupying the upper two-thirds of the right hemithorax with compression of the remaining ipsilateral lung and leftward mediastinal displacement. No pleural effusion or pneumothorax was identified. To further characterize the lesion, contrast-enhanced CT of the chest was performed. Imaging demonstrated a large purely multicystic mass almost completely replacing the right upper and middle lobes. The lesion contained numerous thin-walled cysts of varying sizes without evidence of a solid soft tissue component. No visible communication with the bronchial tree was identified. There was no anomalous systemic arterial supply or abnormal venous drainage. The lesion exerted considerable mass effect with contralateral mediastinal shift and compression of the remaining right lower lobe. No enlarged mediastinal lymph nodes, pleural effusion, chest wall invasion, or distant thoracic abnormalities were observed (Figure 1).

The radiological differential diagnosis included CPAM, cystic pleuropulmonary blastoma, congenital lobar overinflation with secondary cystic change, bronchogenic cysts, and hybrid CPAM-pulmonary sequestration. The absence of a solid component, lack of abnormal systemic vascular supply, and characteristic multicystic morphology favored a diagnosis of CPAM. Given the patient's recurrent respiratory infections, progressive respiratory compromise, and significant mediastinal shift, elective surgical resection was recommended after multidisciplinary discussion involving pediatric surgeons, pediatric pulmonologists, pediatric anesthesiologists, and radiologists.

Following preoperative optimization, including chest physiotherapy and prophylactic intravenous antibiotics, the patient underwent right posterolateral thoracotomy under general anesthesia. Intraoperatively, the right upper and middle lobes were markedly enlarged and replaced by multiple thin-walled cysts of varying diameters, compressing the adjacent lower lobe. No pleural adhesions, abnormal systemic feeding vessels, or communication with the esophagus were identified. A right upper and middle bilobectomy was performed with preservation of the healthy lower lobe. The postoperative period was uneventful. The patient was extubated immediately after surgery and transferred to the pediatric surgical ward following overnight monitoring in the pediatric intensive care unit. Chest tube drainage progressively decreased without evidence of prolonged air leak and was removed on postoperative day 4 after radiographic confirmation of satisfactory expansion of the remaining right lower lobe. Adequate pain control was achieved using multimodal analgesia, and oral feeding resumed on the first postoperative day. Serial chest radiographs demonstrated gradual resolution of mediastinal shift and satisfactory re-expansion of the residual lung.

Gross pathological examination of the surgical specimen revealed multiple interconnected thin-walled cysts ranging from 0.5 to 4.0 cm in diameter, replacing the majority of the excised pulmonary parenchyma. Histopathological analysis demonstrated cysts lined predominantly by ciliated pseudostratified columnar epithelium with focal cuboidal epithelium, separated by delicate fibromuscular septa. No cartilage was identified within the cyst walls, and there was no evidence of immature mesenchymal proliferation, blastematous elements, or malignant transformation. These findings were consistent with CPAM.

The patient was discharged home on postoperative day 7 in stable condition with oxygen saturation of 99% on room air and no signs of respiratory distress. At two-week follow-up, the surgical wound had healed satisfactorily, and respiratory symptoms had resolved completely. Subsequent follow-up visits at 3 and 12 months demonstrated appropriate weight gain, normal developmental progress, and no recurrent respiratory infections or exercise intolerance. Follow-up chest radiography showed satisfactory compensatory expansion of the remaining right lower lobe without recurrent cystic lesions or postoperative complications. The child continued regular surveillance in the pediatric surgery and pulmonology clinics and remained clinically well at the latest follow-up.

## Discussion

CPAM is the most frequently encountered congenital cystic lung lesion and represents a heterogeneous spectrum of developmental abnormalities resulting from disruption of normal tracheobronchial branching morphogenesis during fetal lung development. Although the precise embryological mechanisms remain incompletely understood, aberrant airway branching during the pseudoglandular stage of lung development is considered the principal pathogenic event, leading to replacement of normal pulmonary parenchyma by cystic, dysplastic airways of varying size and complexity [5-8]. CPAM accounts for the majority of congenital pulmonary malformations and has an estimated incidence ranging from approximately 1 in 10,000 to 1 in 35,000 live births [1,3]. The increasing use of routine prenatal ultrasonography has substantially improved antenatal detection; nevertheless, a proportion of lesions remain undiagnosed until infancy or childhood, particularly when prenatal imaging is unavailable or when the lesion is small and initially asymptomatic [2,3].

The clinical manifestations of CPAM are highly variable and depend on lesion size, anatomical location, degree of pulmonary compression, and associated complications [2,5]. Neonates with large lesions may present with severe respiratory distress secondary to mass effect and pulmonary hypoplasia, whereas older infants and children commonly present with recurrent respiratory tract infections, persistent cough, wheezing, or incidental radiographic abnormalities [4,6]. Our patient presented at one year of age with recurrent lower respiratory tract infections and progressive respiratory symptoms, reflecting the natural history of an undiagnosed symptomatic lesion. The marked contralateral mediastinal shift observed on CT indicates substantial mass effect and likely contributed to the patient's respiratory compromise by compressing the remaining functional lung parenchyma [2,5,7]. Such a presentation underscores that significant congenital pulmonary lesions may remain clinically compensated during early infancy before becoming symptomatic with recurrent infection or progressive enlargement.

Cross-sectional imaging remains the cornerstone of postnatal evaluation. While chest radiography provides an initial assessment of lesion location and associated mediastinal displacement, contrast-enhanced CT is considered the imaging modality of choice because it accurately delineates lesion morphology, extent, vascular anatomy, and relationship to adjacent structures [3,4,6]. Identification of anomalous systemic arterial supply is particularly important because it suggests pulmonary sequestration or hybrid lesions and directly influences operative planning. In the present case, CT demonstrated a purely multicystic lesion, replacing nearly the entire right upper and middle lobes without a discernible soft tissue component, bronchial communication, or anomalous vascular supply. These imaging characteristics strongly favored CPAM and were instrumental in narrowing the differential diagnosis while facilitating preoperative surgical planning. Current recommendations advocate postnatal CT evaluation even in prenatally diagnosed lesions to define anatomy and exclude associated vascular anomalies before definitive management [2,5].

The differential diagnosis of large cystic pulmonary lesions in infancy remains broad and includes pulmonary sequestration, hybrid CPAM-sequestration lesions, congenital lobar overinflation, bronchogenic cysts, congenital diaphragmatic abnormalities, and cystic pleuropulmonary blastoma (PPB). Distinguishing CPAM from PPB is particularly important because the latter is a highly aggressive pediatric malignancy that may initially mimic benign congenital cystic lesions on imaging [1,4,6]. Radiological findings favoring PPB include thick septations, mural nodules, a substantial solid component, rapid interval growth, pneumothorax, or evidence of metastatic disease. Conversely, the absence of a solid component, lack of chest wall invasion, absence of abnormal systemic vascular supply, and typical multicystic architecture in our patient supported a benign congenital malformation. Nevertheless, definitive diagnosis relies on histopathological examination after complete surgical excision because imaging findings alone cannot reliably exclude all neoplastic lesions. This emphasizes the importance of multidisciplinary collaboration among pediatric surgeons, radiologists, pathologists, and pediatric pulmonologists in the evaluation of complex congenital lung lesions.

Histopathological assessment remains the diagnostic gold standard and provides important prognostic information. The currently accepted Stocker classification categorizes CPAM into five pathological subtypes according to cyst size, epithelial lining, and presumed level of airway origin [1,3,6]. Type I lesions are characterized by one or more large cysts lined by pseudostratified ciliated columnar epithelium and represent the most common subtype, whereas type II lesions typically contain multiple smaller cysts and are more frequently associated with other congenital anomalies. Types III, IV, and V are considerably less common and differ in both morphology and clinical behavior. In the present case, histopathological findings confirmed CPAM and excluded immature mesenchymal proliferation or malignant transformation, thereby eliminating the principal concern raised during radiological evaluation. Correlation between imaging and pathology is essential because significant overlap exists among congenital pulmonary malformations [2-5].

Management of CPAM continues to evolve, particularly regarding asymptomatic patients. There is broad consensus that symptomatic lesions should undergo complete surgical excision because they are associated with recurrent pulmonary infection, progressive respiratory compromise, pneumothorax, hemorrhage, and, although uncommon, malignant transformation. In contrast, management of asymptomatic lesions remains controversial [4,5,8]. Advocates of elective resection emphasize prevention of infection, facilitation of compensatory lung growth during infancy, elimination of diagnostic uncertainty, and avoidance of potential malignant degeneration. Conversely, proponents of conservative management cite the relatively low incidence of complications in selected patients and the risks inherent to thoracic surgery. Recent international consensus efforts have highlighted persistent variation in clinical practice and emphasized standardized outcome reporting rather than universal agreement on treatment strategy. Nevertheless, symptomatic patients such as ours clearly fulfill accepted indications for operative intervention [1-5].

The extent of pulmonary resection should balance complete removal of abnormal tissue against maximal preservation of healthy lung parenchyma. Anatomical lobectomy remains the most widely accepted surgical procedure because incomplete excision or limited wedge resection may leave residual abnormal tissue and increase the risk of persistent symptoms or recurrence [3-6]. In our patient, the lesion almost completely replaced both the right upper and middle lobes while sparing the lower lobe. Consequently, bilobectomy represented the most appropriate anatomical resection, ensuring complete removal of diseased tissue while preserving the unaffected lower lobe. Although thoracoscopic lobectomy has gained increasing acceptance owing to reduced postoperative pain, shorter hospital stay, and improved cosmetic outcomes, open thoracotomy remains an appropriate approach for giant lesions associated with severe mediastinal displacement, uncertain anatomy, or technical complexity. Ultimately, surgical strategy should be individualized according to lesion characteristics, surgeon expertise, and institutional resources [1-7].

The postoperative course in our patient was favorable, with rapid expansion of the remaining right lower lobe, complete resolution of respiratory symptoms, and satisfactory functional recovery during follow-up [2,6]. These findings are consistent with previous reports demonstrating excellent long-term outcomes following complete resection of CPAM in infancy. The remarkable capacity for compensatory lung growth during early childhood allows preservation of pulmonary function despite anatomical lobectomy or, in selected cases, bilobectomy. Long-term surveillance remains advisable to monitor respiratory function, detect rare postoperative complications, and evaluate overall pulmonary development, particularly in children undergoing extensive resections [1,4,7].

An additional consideration is the potential association between CPAM and malignancy. Although malignant transformation is uncommon, reports describing mucinous adenocarcinoma, adenocarcinoma in situ, rhabdomyosarcoma, and PPB arising within congenital pulmonary malformations have raised concern regarding long-term surveillance and elective resection [2-6]. Molecular studies have identified somatic KRAS mutations in subsets of CPAM, particularly lesions containing mucinous cell clusters, providing biological support for the proposed relationship between some congenital pulmonary malformations and neoplasia. While the absolute risk remains low and is not fully quantified, these observations reinforce the rationale for complete excision of symptomatic lesions and careful pathological examination of all resected specimens [2,5,7].

Our case highlights several clinically important points. First, CPAM should remain an important diagnostic consideration in infants presenting with recurrent respiratory infections or persistent unilateral radiographic abnormalities despite appropriate medical therapy. Second, contrast-enhanced CT is indispensable for defining lesion morphology, assessing vascular anatomy, and guiding operative planning. Third, histopathological confirmation remains essential because imaging findings may overlap with other congenital or neoplastic cystic lung lesions. Finally, timely anatomical resection of symptomatic lesions can relieve mass effect, prevent recurrent infection, establish a definitive diagnosis, and achieve excellent clinical outcomes with preservation of long-term pulmonary function. The successful management of this patient further emphasizes the value of a multidisciplinary approach integrating pediatric surgery, pediatric pulmonology, radiology, pathology, and anesthesiology in optimizing outcomes for children with complex congenital pulmonary malformations.

## Conclusions

This case highlights the importance of considering CPAM in the differential diagnosis of recurrent respiratory infections and persistent unilateral cystic lung lesions in infants, even in the absence of a prenatal diagnosis. Comprehensive radiological evaluation, particularly contrast-enhanced CT, is essential for accurate lesion characterization, assessment of associated vascular anomalies, and preoperative planning, while definitive diagnosis relies on histopathological examination following surgical excision. In symptomatic patients with significant mass effect or recurrent infections, timely anatomical resection provides definitive treatment, relieves respiratory compromise, prevents future complications, and allows excellent functional recovery through compensatory lung growth. Early recognition, multidisciplinary management, and complete surgical excision remain the cornerstones of optimal care, underscoring the need for prompt referral of affected children to specialized pediatric surgical centers to achieve favorable long-term outcomes.
